# The compression type of coronary artery motion in patients with ST-segment elevation acute myocardial infarction and normal controls: a case-control study

**DOI:** 10.1186/1756-0500-4-51

**Published:** 2011-03-07

**Authors:** Aiden JC O'Loughlin, Karen Byth, John K French, David AB Richards, Annemarie Hennessy, A Robert Denniss, Pramesh Kovoor

**Affiliations:** 1 University of Western Sydney, Sydney, Australia; 2 Westmead Millenium Institute, Westmead Hospital, Sydney, Australia; 3 Department of Cardiology, Westmead Hospital, Sydney, Australia; 4 Department of Cardiology, Liverpool Hospital, Sydney, Australia; 5 Department of Cardiology, Blacktown Hospital, Sydney, Australia

## Abstract

**Background:**

Prediction of the location of culprit lesions responsible for ST-segment elevation myocardial infarctions may allow for prevention of these events. A retrospective analysis of coronary artery motion (CAM) was performed on coronary angiograms of 20 patients who subsequently had ST-segment elevation myocardial infarction treated by primary or rescue angioplasty and an equal number of age and sex matched controls with normal angiograms.

**Findings:**

There was no statistically significant difference between the frequency of CAM types of the ST-segment elevation acute myocardial infarction and control patients (p = 0.97). The compression type of CAM is more frequent in the proximal and mid segments of all three coronary arteries. No statistically significant difference was found when the frequency of the compression type of CAM was compared between the ST-segment elevation acute myocardial infarction and control patients for the individual coronary artery segments (p = 0.59).

**Conclusion:**

The proportion of the compression type of coronary artery motion for individual artery segments is not different between patients who have subsequent ST-segment elevation myocardial infarctions and normal controls.

## Introduction

The three-dimensional motion of the heart is characterized by rotation (around the centre of gravity), radial displacement (towards or away from the center of gravity), and translational motion (displacement parallel to its long axis)[1]. The total translational motion of the left ventricle is on average 2.2 cm and is such that motion occurs most at the base and least at the apex of the heart[2].

Motion of individual segments of coronary arteries reflects the motion of the underlying myocardium. The classification system for different patterns of coronary artery motion (CAM) used in this study is derived from a system where CAM was classified into 10 patterns, which were grouped into 3 types: (1) compression type: the length of the arterial segment is shortened without vertical deviation of the artery; (2) displacement type: the location of the coronary artery shifts without change of the length or shape of the arterial segment; and (3) bend type: the coronary artery flexes into a curve[3].

The compression type of CAM for individual artery segments is associated with stenosis[3] and is a predictor of segments containing the culprit lesion responsible for ST-segment elevation myocardial infarctions (STEMIs)[4]. The compression type of CAM has recently been shown to be strongly associated with segments containing the culprit lesion in STEMI patients after successful fibrinolysis[5].

The hypothesis to be tested in this study is that the compression type of CAM is more likely to be present in patients who have subsequent STEMI than in age and sex matched control patients with normal coronary angiograms.

## Methods

Twenty patients were identified who had coronary angiography after March 1998 and subsequently re-presented with a STEMI. STEMI was defined as ischemic chest pain with ST segment elevation of 1 mm in 2 contiguous limb leads or 2 mm in 2 contiguous chest leads. Patients were excluded if they had previous coronary artery bypass surgery or had stent thrombosis as the cause of STEMI. Twenty age and sex matched control patients were identified with normal coronary angiograms.

**Figure 1:**
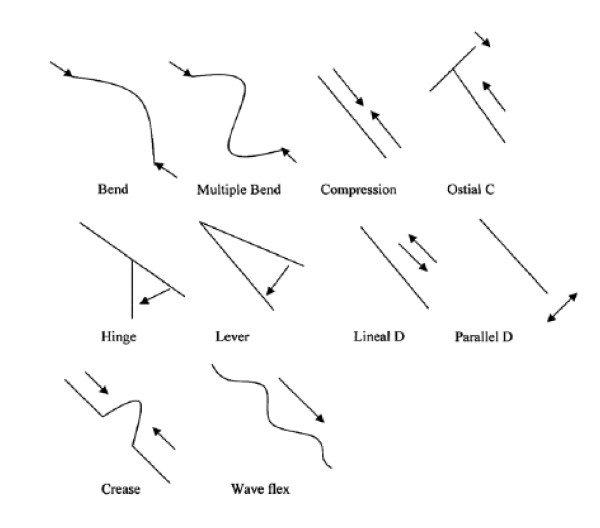
**Classification of coronary artery motion**. Lines illustrate the coronary artery segment and arrows show the direction of coronary artery motion. C = compression, D = displacement.

The CAM patterns of coronary segments were assessed retrospectively in both the STEMI and control patients. For the STEMI patients, the coronary angiography performed before the STEMI was used. The assessment was made blinded to the location of the future culprit segment. The CAM classification of Konta and Bett[3] was used. A schematic of this classification is shown in Figure 1. All three coronary arteries were assessed using all available views. In a single view, a visual comparison was made between the coronary segment at the start and end of systole. A single pattern of motion was assigned in each view. Each segment was then assigned a CAM pattern by synthesizing the assignment for all available views. The patterns of CAM were grouped into the compression type and non-compression type (the bend and displacement types).

**Figure 2:**
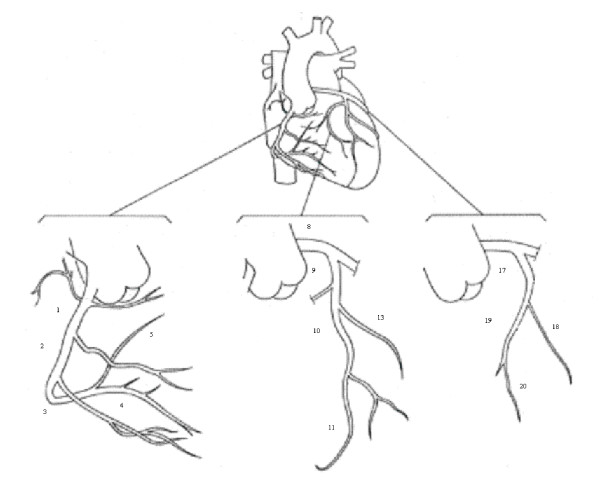
**Coronary artery map**. The figure shows the numerical labeling of segments of the coronary tree.

Assessment of CAM was made in up to fourteen segments of the coronary arterial tree. The segments were given a numerical label as shown in Figure 2.

Clinical risk factors (hypertension, diabetes, smoking, family history and hypercholesterolemia) of all patients were obtained from the medical records.

Chi-squared tests were used for comparison of frequencies between groups. All statistical analyses were performed using Stata (version 10.0, StataCorp, College Station, TX).

The Royal Prince Alfred Hospital Ethics Review Committee approved the research protocol (reference X10-0159). The research protocol did not include obtaining patient consent.

## Results

The demographics for the STEMI and control patients are shown in Table 1. The frequency of each pattern and type of CAM for all the segments of both the STEMI and control patients are shown in Table 2. 67% of segments in both the STEMI and control patients had a non-compression type of CAM. The bend type was present in 44% of segments in the STEMI patients and in 47% of segments in the control patients. The displacement type was present in 23% of segments in the STEMI patients and in 20% of segments in control patients. 33% of segments in both the STEMI and control patients had the compression type of CAM. There was no statistically significant difference between the frequency of CAM types of the STEMI and control patients (p = 0.97).

**Table 1:** Patient Demographics

	STEMI patients	Control patients
Mean age (+/-stdev)	61 (+/-11)	61 (+/-12)

Men (%)	80	80

Diabetes (%)	25	25

Current smoker (%)	45	45

Hypercholesterolemia (%)	75	55

Hypertension (%)	55	50

Family history of coronary artery disease (%)	25	35

**Table 2:** Frequency of each pattern and type of CAM

Pattern of CAM	STEMI patients		Control patients	
	**Number**	**(%)**	**Number**	**(%)**

Non-compression	161	67.1	164	66.9

Bend	106	44.2	114	46.5

Bend	12	5.0	14	5.7

Multiple bend	77	32.1	89	36.3

Hinge	8	3.3	8	3.3

Crease	9	3.8	3	1.2

Wave flex	0	0	0	0

Displacement	55	22.9	50	20.4

Lever	0	0	0	0

Lineal displacement	31	12.9	13	5.3

Parallel displacement	24	10.0	37	15.1

Compression	79	32.9	81	33.1

Compression	71	29.6	65	26.5

Ostial compression	8	3.3	16	6.5

Total	240		245	

**Figure 3:**
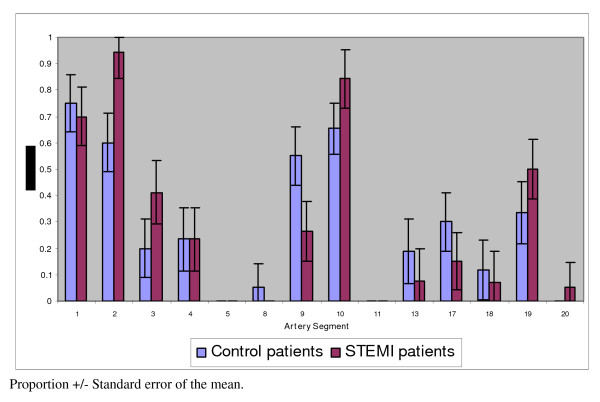
**Proportion of the compression type of CAM for individual artery segments**. The segments in the proximal and mid portions of the three coronary arteries had a high proportion of the compression type of CAM in both control and STEMI patients.

The proportion of the compression type of CAM for individual artery segments for both patient groups is shown in Figure 3. The compression type of CAM was more frequent in the proximal and mid segments of the right (#1 and 2), the left anterior descending (#9 and 10) and to a lesser extent the left circumflex (#17 and 19) coronary arteries. The compression type was less frequent in the distal segments of the right (#3,4, and 5), left main (#8), distal left anterior descending (#11), diagonal (#13), and the obtuse marginal branches (#18 and 20) of the left circumflex coronary arteries. No statistically significant difference was found when the frequency of the compression type of CAM was compared between the ST-segment elevation acute myocardial infarction and control patients for the individual coronary artery segments (p = 0.59).

## Discussion

This study shows that the proportion of the compression type of coronary artery motion for individual artery segments is not statistically significantly different between patients who have subsequent STEMIs and age and sex matched controls.

The main limitations of this study are its small sample size and the potential observer bias in the qualitative assessment of CAM. The technique relies on a visual assessment. Knowledge of the asymmetrical frequency distribution of culprit lesions in patients with STEMIs[6] and the presence of stenosis within a segment may bias the qualitative visual assessor.

**Figure 4:**
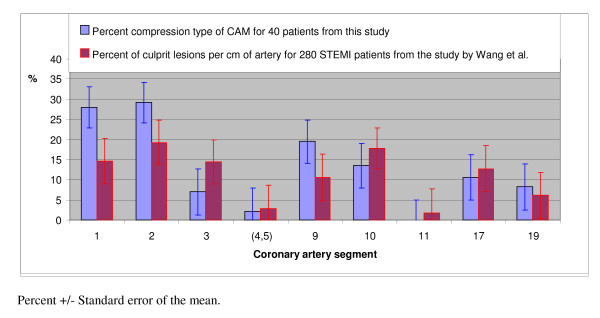
**Comparison of segmental distributions of STEMI and the compression type of CAM**. The percentage of culprit lesions per centimeter of artery was calculated from the data for 280 STEMI patients in the study by Wang et al[6]. The percentage of compression type of CAM per centimeter of artery for the 40 patients of this study was calculated by dividing the proportion of the compression type of CAM for the segment by its length in centimeters.

Although the exact pattern of CAM varies amongst individual patients, there are consistent themes of motion differences between the different coronary arteries[7,8] and along individual arteries[9]. The coronary segments that had high proportions of the compression type of CAM have previously been shown to include the site of most STEMIs[6,10]. The distribution in coronary segments of the compression type of CAM for the 40 patients in this study and the location of the culprit lesion responsible for STEMI in 280 patients in a previously published report[6] are shown in Figure 4. The percent compression type of CAM and the percent of culprit lesions per cm of artery was highest for the proximal and mid parts of the right coronary artery (segments 1 and 2) and the proximal and mid parts of the left anterior descending coronary artery (segments 9 and 10).

Previously published work has shown that the compression type of CAM is a predictor of the location of stenosis[3] and the culprit segment responsible for STEMI[5,11]. This study builds on this work by finding no difference in the proportion of the compression type of coronary artery motion between the STEMI population and normal controls. A possible explanation for these findings is that systemic factors determine whether a patient develops coronary atherosclerosis and local biomechanical and/or haemodynamic shear stress determines its location within the coronary arteries.

## Competing interests

The authors declare that they have no competing interests.

## Authors' contributions

AO and PK conceived the study. AO undertook the ethics application, data collection, data analysis, and manuscript preparation. KB recommended the statistical methods and supervised data analysis. JF, DR, AH and RD prepared the ethics application, performed manuscript revision and supervised the activities of AO. All authors except KB read and approved the final manuscript.
